# Interest In and Uptake of HIV Pre-Exposure Prophylaxis (PrEP): A Cross-Sectional Study of High-Risk Patients in Western Canada

**DOI:** 10.7759/cureus.24279

**Published:** 2022-04-19

**Authors:** Stanley Enebeli, Udoka Okpalauwaekwe, Prosanta K Mondal, Johnmark Opondo, Anne Leis

**Affiliations:** 1 Community Health and Epidemiology, University of Saskatchewan, Saskatoon, CAN; 2 Academic Family Medicine, University of Saskatchewan, Saskatoon, CAN; 3 Clinical Support Research Unit, University of Saskatchewan, Saskatoon, CAN; 4 Public Health Services, Saskatchewan Health Authority, Saskatoon, CAN

**Keywords:** canada, sexual behaviour, prep awareness, hiv/aids, hiv risk perception, prep interest, pre-exposure prophylaxis

## Abstract

Rationale

Pre-exposure prophylaxis (PrEP) is a highly effective, evidence-based HIV prevention strategy. However, its use in the city of Saskatoon, Saskatchewan province of western Canada, is relatively new. Therefore, this study aimed to examine the interest and uptake of PrEP and investigate factors associated with HIV PrEP by high-risk patients.

Methods

A cross-sectional, self-administered survey of patients attending Saskatoon’s Public Health Services Sexual Health Clinic was conducted from October until December 2018. The primary outcome was the interest in taking PrEP to reduce the risk of HIV infection. This outcome was evaluated for its association with potential correlates, which included: sociodemographic characteristics, HIV risk perception, prior PrEP awareness, and sexual behaviors/lifestyles. Descriptive, univariate, and multivariate analyses were used to pursue our research objectives.

Results

One hundred forty-one participants were recruited from a sexual health clinic in Saskatoon. The median age (interquartile range) was 26 (22-31) years. The median number of partners (interquartile range) was 3 (2-4) partners. A total of 66.0% of participants were unaware of PrEP, and almost half ( 49.6%) indicated an interest in taking PrEP. Among those disinterested in PrEP, 49.3% perceived minimal HIV risk, 35.2% expressed concern regarding side effects, 28.1% cited the added need for condom use, 23.9% indicated incomplete effectiveness, and 22.5% did not wish to undergo regular bloodwork. Multivariate analysis showed that interested patients were more likely to have been previously aware of PrEP (OR: 2.6, p-value = 0.03), perceived themselves to be vulnerable to HIV (OR: 15.7, p = <0.0001), or were unsure about their risk (OR: 3.9, p = 0.001).

Conclusion

This study suggests that a lack of knowledge regarding personal HIV risk and PrEP as a preventive option may influence PrEP interest. There lies a need for more health promotion campaigns around the health benefits of PrEP, including literacy efforts on HIV risk, concerns around side effects, and associated blood work with PrEP use.

## Introduction

HIV infection persists as a significant public health problem impacting more than 70 million people globally [1]. In addition, new infections occur every year, emphasizing the need for more integrated HIV prevention programs and strategies [1, 2]. Pre-exposure prophylaxis (PrEP) is a highly effective, evidence-based HIV prevention strategy [3]. PrEP involves taking daily oral antiretroviral (ARV) medications such as tenofovir disoproxil fumarate/emtricitabine (TDF/FTC) or tenofovir alafenamide combined with emtricitabine, on an ongoing basis, before and after potential exposure to HIV [4]. In Canada, the use of TDF/FTC for PrEP was approved by Health Canada in February 2016, and in July 2017, lower-cost generic versions were made available nationwide [5]. Since its approval, PrEP has been integrated into HIV prevention strategies in Canada, primarily focusing on bisexuals and men who have sex with men (MSM) [6, 7]. The Canadian guideline on HIV PrEP provides clinical criteria for using this intervention among those at high risk for HIV infection, including MSM. This high-risk group accounts for almost half of newly diagnosed cases of HIV infection nationwide [1, 8, 9].

Despite the potential for PrEP to be an effective HIV prevention strategy, its acceptance and uptake have faced some challenges [7, 10]. As of 2012, only five countries had formally adopted PrEP, which included the United States, France, South Africa, Kenya, and Canada [11]. However, seven countries (Peru, Ukraine, India, Kenya, Botswana, Uganda, and South Africa) were willing to adopt PrEP among key populations [10]. Recent studies have shown that a lack of awareness of PrEP makes up a significant barrier to accessing preventive treatment [11-16]. Although awareness of PrEP in Canada appears to have increased over time [17], studies show varied reports on the knowledge of and attitudes to PrEP across high-risk communities and other settings [17-21]. A 2016 Vancouver study by Knight R et al. showed that awareness of PrEP was generally low in those at high risk of HIV infection [20]. A 2014 Vancouver survey reported that 21% of HIV-negative gay, bisexual, and other men who have sex with men (GBMSM) were aware of PrEP [18], while a survey of the GBMSM community in Ottawa found that 53% of participants knew about PrEP [17]. A survey-based study in Toronto among the GBMSM community showed that awareness of PrEP had increased from 14% in 2010 to 72% in 2015 [19]. Furthermore, a recent survey among young sexual minority men and transgender women in New York City revealed that while 98% of respondents were aware of PrEP, only 23.2% were taking it [21].

Several other studies have reported barriers to the uptake and retention of PrEP by at-risk populations, including PrEP side effects, PrEP effectiveness, PrEP compliance once started, including daily dosing, and bloodwork frequency [17, 20, 22, 23]. In addition, structural level barriers, including health practitioners' awareness and willingness to provide PrEP and necessary follow-up care [24], cost of PrEP for non-generic versus generic brands [25], absence of funding schemes [25], lack of third-party insurance [25], and stigmatization around HIV, especially among ethnic communities like blacks, Hispanics and people of color (POC) [17, 20], have also been reported to contribute to the uptake and retention of PrEP.

Context

Saskatchewan Province and the Healthcare System

Saskatchewan is one of the 13 provinces and territories in western Canada, with over 1 million in population, half of which live in major cities like Saskatoon and Regina [26]. Saskatchewan is known for its unique population distribution (urban, metropolitan, reserves, rural municipalities, villages, hamlets, and crown colonies), including its relatively sizeable indigenous population [26]. Saskatchewan offers a publicly funded healthcare system where almost all medically necessary hospital and physician services are delivered free or at supplemental coverages [27]. In addition, the government provides complete cost coverage for PrEP in Saskatchewan [28].

HIV in Saskatoon

Saskatoon is an ethnically diverse city with approximately 300,000 residents [29]. In 2016, Saskatoon's HIV rate was two times the national rate (12.5 vs. 5.8/100,000), according to the Saskatoon Health Region HIV Special Report [30]. The primary routes of HIV transmission reported were injection drug use (IDU) at 63%, heterosexual sex at 23%, and MSM at 11% [30].

Study rationale

As far as we know, no study on PrEP interest, acceptance, and retention has been carried out in Saskatchewan. Therefore, this study examines the factors associated with interest in PrEP in a sample of HIV-negative, at-risk individuals in Saskatoon, Saskatchewan.

## Materials and methods

Ethical considerations

This research was reviewed and approved by the University of Saskatchewan Behavioral Research Ethics Board (Beh #322). Operational approval was also granted by the Saskatchewan Health Authority (SHA).

Study design and sampling technique

A cross-sectional study design informed the processes of this study. We employed a non-probability (convenience) sampling technique to recruit participants for this study. Our targeted sample size was 123 based on mathematical computations posited by Peduzzi P et al. [31] for sample size calculations involving binary logistic regression analyses.

Setting and participant recruitment

This study was based out of a sexual health clinic operated by Public Health Services in Saskatoon. This walk-in clinic provides care to a broad age group, including adolescents and seniors, focusing primarily on sexual and reproductive health concerns. An average of 270 clients are seen in this clinic monthly. All individuals who visited the clinic between October 24, 2018, and December 07, 2018, were invited to participate in this study. Recruitment was facilitated by the lead author, who approached individuals during clinic hours seeking consent to participate in this study. During this time, the study objectives were explained to potential participants, and screening for eligibility was carried out before consent was obtained. Inclusion criteria constituted individuals who were 18 years and were sexually active with either of the following sexual preferences: MSM; bisexual male or female; had been with more than two sexual partners in the last six months; reported infrequent condom use (reported subjectively as never, sometimes or usually); reported IDU in the past six months; were HIV negative. Exclusion criteria were individuals under 18 years; individuals 18 years and older who were sexually inactive, or if sexually active, always used a condom with only one partner; had engaged sexually with known HIV-positive individuals; did not engage in IDU; were known, HIV-positive individuals. Consenting participants who met the inclusion criteria were handed a survey questionnaire to fill out by themselves in the clinic and return on completion the same day.

Data collection

**Figure 1:**
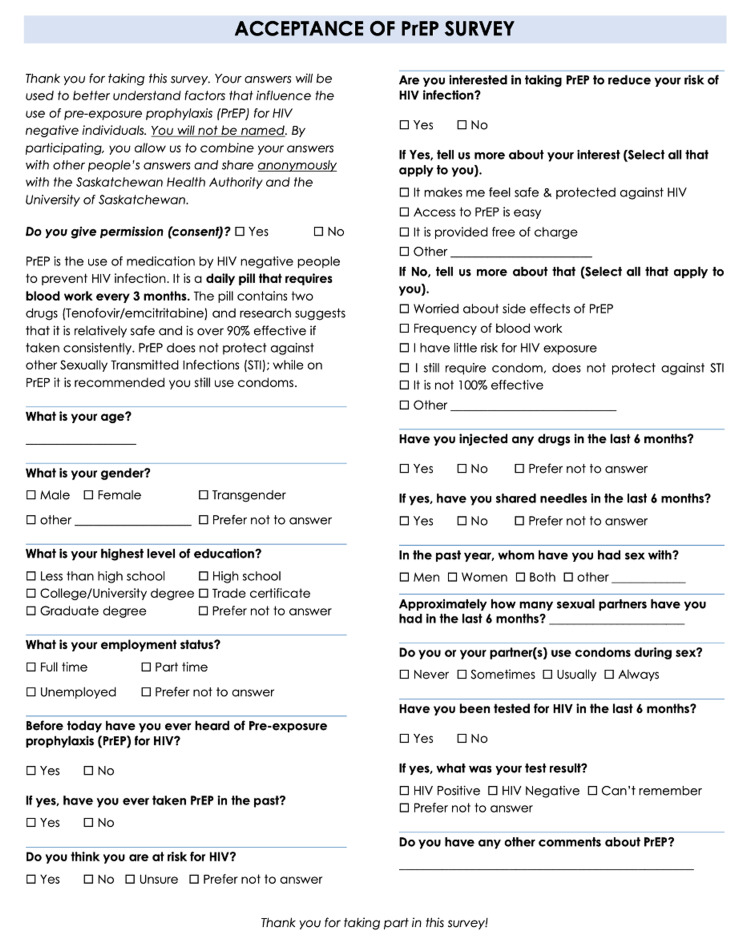
Study survey instrument.

Data were collected using a paper-based, self-administered questionnaire developed by the research team in collaboration with the Public Health Observatory (PHO), Population and Public Health, Saskatoon. This questionnaire was pilot tested on clinic patients for clarity and face validity, revised based on feedback, and reviewed again by public health experts before field testing. See appendices (Figure 1) for a sample of the survey instrument.

Measures 

Awareness of PrEP

To evaluate their awareness of PrEP, participants were asked on the survey the question, "Before today, have you ever heard of pre-exposure prophylaxis (PrEP) for HIV?" to which they could respond with a "yes" or "no." A "yes" response was considered as being aware of PrEP. 

Interest in PrEP

To evaluate the interest of participants in taking PrEP, the question "Are you interested in taking PrEP to reduce your risk of HIV infection?" was posed to participants, to which they could respond with a "yes" or "no." A "yes" response was considered as being interested in taking PrEP. 

Socio-demographic characteristics

Self-reported demographic characteristics included the following with corresponding options: age, gender (male, female, other, or prefer not to answer), the highest level of education (less than high school, high school, college/university degree, trade certificate, graduate degree, or prefer not to answer), employment status (full-time, part-time, unemployed, or prefer not to answer).

Risk perception of HIV

Risk perception was assessed with the question, "Do you think you are at risk for acquiring HIV infection?" Participants could respond with a "yes" or "no" or "unsure" or "prefer not to answer."

Sexual behavior and lifestyle

Variables in this category included MSM, bisexual male or female, number of sexual partners in the last six months, condom use during sex, IV drug use in the last six months, and a number of sexual partners within the last 12 months. 

Outcome variables

The outcome variable for this study was "interest in PrEP," categorized as a binary variable with responses as yes or no.

Potential correlates

Potential correlates were awareness of PrEP, socio-demographic characteristics, risk perception of HIV, and sexual behavior and lifestyle.

Statistical analysis

Data analysis was carried out using IBM SPSS Statistical software version 27.0 (IBM Corp., Armonk, New York, USA). Preliminary descriptive statistics (medians with 25th and 75th quartiles, frequencies with percentages) were initially calculated to characterize the sample. The regression analysis was conducted in a two-step process. First, Chi-squared tests (or Fisher's exact test where expected cell values were less than 5) were used for bivariate comparisons. The level of significance (alpha) was set at 0.05 for two-sided statistical tests. Significant variables at the bivariate level (i.e., p-value <0.05) were entered into the multivariate logistic regression model using the default forced entry method to estimate the OR and 95% CI for the outcome variable and interest in PrEP. Variables removed were also tested to determine if they were confounding variables.

## Results

A total of 382 patients were invited and screened for inclusion during the study period. Out of this number, 311 met inclusion criteria. However, only 141 returned completed surveys. Table 1 summarizes the descriptive characteristics of overall study variables and univariate analyses results (p-values), categorized by the individuals interested in PrEP and individuals not interested in PrEP. Overall, 66% (n=93) of respondents were unaware of PrEP, and 50.4% (n=71) were not interested in taking PrEP to reduce the risk of HIV infection. Interest in PrEP was univariably and statistically associated with awareness of PrEP, risk perception of HIV, MSM, and the number of sexual partners in the last six months. 

**Table 1:** Summary of descriptive and univariate analyses of predictor variables and the outcome of interest in PrEP. IDU: Intravenous drug use; MSM: Men having sex with men; PrEP: Pre-exposure prophylaxis. *p-value significant at 0.05 level.

Variables	Categories	Total (n=141)	Interest in PrEP (n=70)		No interest in PrEP (n=71)	
		Frequency (%)	Frequency (%)		Frequency (%)	P-value
Socio-demographics						
Age (in years)	Median (25, 75)	26 (22, 31)	26 (22, 33)	27 (21, 30)		0.715
Gender	Female	55 (39.0)	22 (31.4)	33 (46.5)		0.077
	Male	83 (58.9)	46 (65.7)	37 (52.1)		
	Other	3 (2.1)	2 (2.9)	1 (1.4)		
Level of education	Less than high school	4 (2.8)	2 (2.9)	2 (2.8)		0.357
	High school	54 (38.3)	28 (40.0)	26 (36.6)		
	College/University degree	56 (39.7)	31 (44.3)	25 (35.2)		
	Trade certificate	13 (9.2)	4 (5.7)	9 (12.7)		
	Graduate degree	12 (8.5)	4 (5.7)	8 (11.3)		
	Prefer not to answer	2 (1.4)	1 (1.4)	1 (1.4)		
Employment	Full-time	78 (55.3)	40 (57.1)	38 (53.5)		0.717
	Part-time	27 (19.1)	12 (17.1)	15 (21.1)		
	Unemployed	29 (20.6)	16 (22.9)	13 (18.3)		
	Prefer not to answer	7 (4.9)	1 (1.4)	6 (8.4)		
Awareness of PrEP						
Have previously heard of PrEP	Yes	48 (34.0)	34 (48.5)		14 (19.7)	<0.0001*
	No	93 (66.0)	36 (51.4)		57 (80.2)	
Risk perception of HIV						
Do you think you are at risk for HIV?	Yes	23 (16.3)	21 (30.0)		2 (2.8)	<0.0001*
	No	63 (44.7)	17 (24.3)		46 (64.8)	
	Unsure	51 (36.2)	29 (41.4)		22 (31.0)	
	Prefer not to answer	4 (2.8)	3 (4.3)		1 (1.4)	
Sexual behaviors						
MSM	Yes	35 (24.9)	28 (40.0)		7 (9.8)	<0.0001*
	No	102 (72.3)	40 (57.1)		62 (87.3)	
	Prefer not to answer	4 (2.8)	1 (1.4)		3 (4.2)	
Bisexual	Yes	14 (10.0)	8 (11.4)		6 (8.4)	0.554
	No	123 (87.2)	60 (85.7)		63 (88.7)	
	Prefer not to answer	4 (2.8)	2 (2.9)		2 (2.8)	
IDU in last 6 months	Yes	5 (3.5)	4 (5.7)		1 (1.4)	0.111
	No	133 (94.3)	65 (92.9)		68 (95.8)	
	Prefer not to answer	3 (2.1)	1 (1.4)		2 (2.8)	
Number of sexual partners in last 6 months	Median (25, 75)	3 (2, 4)	4 (2, 5)		3 (2, 4)	0.013*
Condom use during sex	Never	19 (13.5)	10 (14.3)		9 (12.7)	0.222
	Sometimes	63 (44.7)	31 (44.3)		32 (45.1)	
	Usually	40 (28.4)	21 (30.0)		19 (26.8)	
	Always	19 (13.5)	8 (11.4)		11 (15.5)	

Table 2 describes the frequency and percentages of reasons for interest or disinterest in PrEP. The most common reasons for interest in PrEP among interested participants were the sense of protection and safety (90%), getting PrEP free of cost (57.1%), and easy access to PrEP (42.9%). Conversely, the most common reasons for non-interest in PrEP among non-interested respondents were low self-perception of risk for HIV (49.3%), worry about side effects (35.2%), and the added requirement to use condoms against sexually transmitted infections (STIs) (28.1%).

**Table 2:** Frequencies of reasons for interest and non-interest in PrEP. PrEP: Pre-exposure prophylaxis; STI: Sexually transmitted infection. ^a^Respondents may have indicated multiple reasons.

Interested in PrEP (n = 70)		Not interested in PrEP (n = 71)	
Reason^a^	n (%)	Reason^a^	n (%)
It makes me feel safe and protected against HIV	63 (90.0)	I have little risk for HIV exposure	35 (49.3)
It is provided free of charge	40 (57.1)	Worried about side effects of PrEP	25 (35.2)
Access to PrEP is easy	30 (42.9)	I still require condom, does not protect against STI	20 (28.1)
		It is not 100% effective	17 (23.9)
		Frequency of blood work	16 (22.5)

Table 3 shows the results of the multivariate analyses for predictive factors associated with interest in PrEP (i.e., awareness of PrEP, risk perception of HIV, MSM, and the number of sexual partners in the last six months from the univariate analyses in Table 1). MSM and awareness of PrEP showed a high correlation (multicollinearity), resulting in the exclusion of MSM from the final model (Table 3). Hence, estimates from the adjusted model indicated that participants who gave a "yes" response to the question, "do you think you are at risk for HIV" were 15.7 times more likely to be interested in taking PrEP to reduce their risk of HIV infection. Likewise, participants who gave an "unsure" response to the question, "do you think you are at risk for HIV" were 3.9 times more likely to be interested in taking PrEP to reduce their risk of HIV infection. Awareness of PrEP for HIV prophylaxis was 2.5 times associated with a likelihood of showing interest to take PrEP to reduce their risk of HIV infection.

**Table 3:** Multivariate associations between potential correlates and interest in PrEP. PrEP: Pre-exposure prophylaxis; Ref: reference group.

Covariates	Interest in PrEP	
	OR (95% CI)	P-value
Do you think you are at risk for HIV?		
No	Ref	
Yes	15.71 (3.11-50.04)	<0.0001
Unsure	3.99 (1.73-7.86)	0.001
Awareness of PrEP		
No	Ref	
Yes	2.56 (1.10-6.10)	0.03
Number of sexual partners in last 6 months	1.11 (0.98-1.35)	0.29

## Discussion

This study investigated interest in the uptake of PrEP among at-risk HIV-negative adults in Saskatoon. Our study sample had 83% identified as male, with a median age of 26 years. A total of 97% of participants had continued their education beyond high school, with 74.5% having full or part-time employment. One in four participants identified as MSM, and only 16% of respondents believed that they were at risk for HIV acquisition.

Our study showed that 66% of our respondents had not heard of PrEP before participation in this survey, and more than half of the participants indicated no interest in PrEP. However, respondents with prior awareness of PrEP were more likely to be interested in PrEP. These findings are consistent with prior studies in Canada and other countries, which showed relatively low awareness and interest in PrEP [17, 18, 20, 22-24, 32,33].

Our study showed that a lack of PrEP awareness prior to participation in our survey was significantly associated with disinterest in taking PrEP. Therefore, raising more awareness and literacy regarding PrEP, especially in high-risk individuals, may more likely be an obvious first step in growing their interest and use of PrEP. In addition, although socio-demographic characteristics (i.e., age, gender, level of education, and employment) were not significantly associated with interest in PrEP in our regression analyses, studies have shown a higher level of education and employment to be positively correlated with awareness of PrEP [7, 34].

Significantly related to interest in PrEP was a perception of risk for HIV. Our study revealed that individuals who believed themselves to be at little risk of HIV exposure were much less likely to be interested in PrEP. Comparable with outcomes from some US-based studies [25, 35], this worrisome finding calls for a crucial need for further inquiry, as 45% of our study respondents felt they were at little risk of HIV infection despite being a part of this high-risk group. Additionally, our sample also had a considerable proportion of individuals who were uncertain about their risk (37%) and appeared concerned about it, given their increased interest in PrEP over those perceiving no risk. Refortified and targeted health promotion strategies put forward on an individual and community level have been demonstrated to benefit individuals in this population as they are positioned to learn more about their risk for HIV [36]. While it is important to promote the uptake of PrEP, similar efforts should also be provided, to those who have initiated PrEP to avoid negative compensatory behaviors, like the practice of sex without condoms, as this behavior is not routinely and expressly evaluated among at-risk groups. This is important because PrEP does not protect against other STIs, hence the need for safe sex promotion strategies [37].

Concerns regarding side effects were the second most cited reason for disinterest in PrEP, which is consistent with similar studies [17, 20, 22]. PrEP has been validated as generally safe and well-tolerated by most individuals [3]. Common side effects of PrEP have been classified as mild to include headache, nausea, diarrhea, and fatigue [9, 17, 38]. However, it is essential and reassuring to at-risk individuals when their primary care provider or public health professional considers this barrier as pertinent to them enough to seek out measures to mitigate against deteriorating or worsening side effects. Identifying and minimizing barriers to PrEP commencement is beneficial, albeit partial. Ongoing therapy is necessary and should not be assumed, as research shows that PrEP is most effective if taken daily and consistently [8, 9, 38].

An interesting finding from our study was the incremental association between the number of sexual partners and the interest in PrEP during our model building for outcome interest in PrEP. This association appeared to be driven by risk perception of HIV, supported by the loss of significance when risk perception was added to the multivariable model. Thus, our findings suggest that there may be good basic knowledge about perceived HIV risk with an increasing number of sexual partners. In addition, among individuals interested in PrEP in our study, 57% and 43% indicated that their interest was related to the medication being free of charge and easy to access, respectively. This suggests that eligible individuals, even those interested, may not have realized that the medication is available without cost and may require assistance to receive it. 

Our study highlights the need for continued efforts to promote the awareness and uptake of PrEP among at-risk populations in Saskatoon, Saskatchewan, and across Canada. We believe the findings from this study can provide a foundational and additive basis for creating policies and developing strategies that would promote the uptake and retention of PrEP in Saskatchewan, Canada. Granted, there have been significant strides in advancing the uptake and retention of PrEP across Canada [8, 17, 38]; however, there remains the need for renewed and re-calibrated efforts to facilitate fast-track targets towards Project 2030 [39, 40].

Study limitations

Some significant limitations need to be considered when interpreting our study results. Firstly, caution should be exercised when applying these study results to the entire Saskatchewan population, as our study sample was limited to Saskatoon and not stratified to reflect the knowledge, opinions, and behaviors of at-risk people elsewhere. We notably lacked the inclusion of indigenous communities in Saskatchewan, where HIV rates have often been higher than that of the non-indigenous population in Saskatchewan [30]. Secondly, data were self-reported by respondents in this study. This may have resulted in under-reporting of the number of partners, a potential source of bias if preferentially occurring among those not interested in PrEP. Seven participants indicated previous use of PrEP; as such, these results do not purely reflect the perspectives of individuals who have not been personally introduced to this preventive option. Current use was also not assessed. Our response rate was under 50%, so selection (volunteer) bias may also be present if those who chose to respond are not representative of the total at-risk population attending the sexual health clinic. Finally, although survey materials were explicit that only HIV-negative people are eligible for PrEP, and we did inquire about positive HIV testing within the last six months, it is possible that a small number of individuals with an unknown diagnosis of HIV may have completed the survey.

## Conclusions

Saskatoon's at-risk population for HIV frequently includes young adults with multiple partners, limited condom use, and low awareness of their vulnerability to infection. Our study suggests that a lack of knowledge regarding personal HIV risk and PrEP as a preventive option may influence PrEP interest. Effective mitigation of respondent concerns around side effects and bloodwork may improve uptake, as may ensure that eligible individuals can access the medication and are aware of full cost coverage in our provincial setting.

## Appendices

Supplemental file 1
